# The Real Test of the Nursing Spirit

**Published:** 1919-05-31

**Authors:** 


### THE MATRONS' AND SISTERS' DEPARTMENT.

### THE REAL TEST OF THE NURSING SPIRIT.

It is very significant to note in the lay papers how widespread is the recognition among members of the public that Edith Cavell's spirit was that principle of love to which nurses dedicate their lives. She is hailed everywhere as the true type of nurse. Every one who knew nurses recognised in her actions and mind the realisation of something which they had seen nurses to be striving for. The fragrance and strength of her memory hold something supernatural, the essence of that vocation which triumphs easily over the manifestations of brute force, triumphs (though hardly) over the human forces of cunning, triumphs with supreme surrender, even over the erring impulses of self.

Seldom has it happened that a hidden life filled with monotonous duties has been so dramatically drawn into the blaze of publicity. But every fact relating to Edith Cavell reveals her more clearly as the nurse we would all aim at imitating. Her heroic deeds are displayed as the fruitage of a life spent like that of thousands in concentration on details, and in the patient moulding of other lives in accordance with a hidden pattern. Like so many of those whose heroism in camp and clearing station has been recounted in these columns, she took very small account of the brave things she was called on to do. Death had no terrors for her. She said it was too familiar. Saving the lives of prisoners and fugitives came as natural to her as to care for the wounded. She troubled as little about the risks she ran as about the risk of infection from a fever patient. And this quiet, unconscious courage is recognised by the public to-day as characteristic of her profession.

What Miss Cavell was exercised about in her last hours was that her every thought should be brought into conformity with the ideals of her whole life. "Patriotism is not enough." Patriotism was a joy. It came easy to her. She must look deeper into the springs of her nature, and before she surrendered her soul the faintest trace of hatred or bitterness must be expunged. Thus at the end she nursed no rankling thought of her services to the German wounded and the base ingratitude with which they had been repaid, no perception of her own outraged dignity as an Englishwoman, no detestation of her enemies, no contempt for the judge who hurried on her execution. "She nothing common *thought* nor mean, Upon that memorable scene." Already her life had passed beyond the veil and Love had triumphed over baser preoccupations.

The spirit of Miss Cavell is what the nursing profession needs in this hour, and perhaps it is not too much to say, it is the spirit the nursing profession already begins to possess. It is the spirit which cares intensely about the organisation of nursing and the character of those who are to carry on its traditions. It is the spirit which believes that certain basic principles of right and wrong are worth fighting for to the death, which swerves from no sacrifice in following its star, and will have no lot or part with railing or rancour. The greatest engine of tyranny the world has ever seen, the German military administration, was faced with this spirit and it proved a touch-stone to show mankind how unutterably paltry and feeble a thing cunning and malice, when raised to their highest powers, could produce.

The evils which war has brought in its train are varied and gigantic enough to dismay the boldest. It is on the nursing profession that the hardest part of the battle against disease and social evils will be thrown, for it is nurses who will have to clear up the details and deal with individual lives. It is well they should be nerved in this first realisation of the labours which peace promises by proof of the irresistible power of their vocation.

The nursing profession is a finer conception than any one yet guesses. The seed of greatness within it has already fallen on good ground and borne fruit for the solace of humanity. But its future lies hazy before it. Whether it will be wrecked on the rock of bitterness and barren controversy, or whether, united within itself and upborne on a strong spirit of mutual confidence, it will have power to reorganise itself under a changed condition of society, is now to be decided. There must be no easy compromise. There must be no paltering with right and justice for the sake of peace at any price. There must be no gambling with the future in the hope that after all things will come right in the end. But there must be no bitterness. Loyalty is not enough. Where rancour lingers there is the seed of decay.

